# Cargo Transport by Cytoplasmic Dynein Can Center Embryonic Centrosomes

**DOI:** 10.1371/journal.pone.0067710

**Published:** 2013-07-01

**Authors:** Rafael A. Longoria, George T. Shubeita

**Affiliations:** 1 Center for Nonlinear Dynamics and Department of Physics, The University of Texas at Austin, Austin, Texas, United States of America; 2 Institute for Cellular and Molecular Biology, The University of Texas at Austin, Austin, Texas, United States of America; University of Colorado, Boulder, United States of America

## Abstract

To complete meiosis II in animal cells, the male DNA material needs to meet the female DNA material contained in the female pronucleus at the egg center, but it is not known how the male pronucleus, deposited by the sperm at the periphery of the cell, finds the cell center in large eggs. Pronucleus centering is an active process that appears to involve microtubules and molecular motors. For small and medium-sized cells, the force required to move the centrosome can arise from either microtubule pushing on the cortex, or cortically-attached dynein pulling on microtubules. However, in large cells, such as the fertilized *Xenopus laevis* embryo, where microtubules are too long to support pushing forces or they do not reach all boundaries before centrosome centering begins, a different force generating mechanism must exist. Here, we present a centrosome positioning model in which the cytosolic drag experienced by cargoes hauled by cytoplasmic dynein on the sperm aster microtubules can move the centrosome towards the cell’s center. We find that small, fast cargoes (diameter ∼100 nm, cargo velocity ∼2 µm/s) are sufficient to move the centrosome in the geometry of the *Xenopus laevis* embryo within the experimentally observed length and time scales.

## Introduction

A common feature of many eukaryotic cells is that the centrosome, the organelle that acts as the main microtubule organizing center, is positioned and maintained at, or close to, the geometric center of the cell during interphase [1]–[6]. Centering the centrosome is an active process that involves cytoskeletal and molecular motor proteins [1]–[3], [5]–[14]; however, the precise role each one of these proteins plays is not fully understood and may differ in different cell types. In smaller cells, microtubule pushing on the cell cortex can create enough force to move the centrosome [15]. The forces generated can be as large as tens of piconewtons [10], [16], enough to generate motion in the crowded cytoplasm. The centrosome in fission yeast has been shown to be centered by such microtubule pushing [12], [13]. This mechanism however, is limited by the mechanical stiffness of the microtubules. The buckling force for a microtubule decreases as the microtubule length increases [6], [10], [15], [16], and thus, for larger cells a different mechanism must exist. Microtubules can act as a tether connecting the centrosome to cortical motors that pull the centrosome towards the cortex as the motor proteins translocate along the microtubules [6], [8], [9]. At first sight, this mechanism would appear to decenter the centrosome since microtubules will touch the cortical side closer to the centrosome before microtubules reach the opposite cell boundary. A simple solution to this problem was proposed by Grill and Hyman [8]: if the cortical motors are equally distributed over the cell cortex and their number is limited, i.e. there are less cortical motors available than microtubules reaching the cortex, a simple geometric analysis shows that there will be more cortically-anchored microtubules producing a force towards the cell center than those pulling the nucleus towards the near cortical side. Indeed, pulling forces can be responsible for centering in mammalian cells [1], the *C. elegans* embryo [8], [9] and budding yeast [17]. This mechanism however, requires that microtubules reach the far cortical side of the cell before the centrosome can start moving to the center. In larger cells, such as the fertilized *Xenopus laevis* embryo (diameter ∼1200 µm), the male pronucleus, together with its associated centrosome, begin their motion towards the center before microtubules reach the periphery on the far cortical side [6]. Furthermore, the *Xenopus laevis* embryo is too large for microtubules to generate enough pushing force to move the pronucleus without significant buckling. Bundled or crosslinked microtubules can withstand much larger forces before buckling and thus could potentially play a role in centrosome centering. However, at least in *Xenopus laevis* embryos, reinforced microtubule networks have not been experimentally observed, and the available experimental evidence argues against the existence of a stiffened microtubule network [6]. Thus, microtubule pushing is likely to play only a minor role in centrosome centering. Previous works suggested cytoplasmically distributed forces are responsible for pronucleus motion [18]. If motor proteins are cytoplasmically distributed, rather than cortically bound, the number of motors that can attach to a microtubule increases with microtubule length. Thus, more motors will pull on the microtubules extending into the far cortical side since those can elongate unobstructed, and the net resulting force on the sperm aster will point towards the cell center. The question of how cytoplasmically distributed motors can transmit a force to the centrosome through the microtubular network has recently gathered much interest [2], [3], [6], [11]. In some cases, it has been argued that relatively fixed structures within the cell act as anchors for the cytoplasmically distributed motors [6], [10], [19]. However, while in flat cells cortical motors could engage microtubules along their lengths and lead to a similar effect as that expected from cytoplasmically-distributed motors [14], no such fixed structures are known experimentally in non-flat cells. The possibility that microtubule-based moving cargoes can act as load-bearing anchors has recently been investigated [2], [3], [11]. Conceptually, this mechanism is simple: a cargo moving through the cytoplasm experiences an opposing drag force which has to be matched by the motors pulling it. This force is transmitted to the microtubule on which the motors are hauling the cargo along, effectively pulling on the microtubule and associated structures, e.g. centrosome and pronucleus, in the direction opposite to the motion of the cargo. Indeed, several different cargoes (yolk granules, lysosomes, endosomes, etc.) are known to be transported along microtubules by dynein during centrosome centering [3], [7]. In *C. Elegans* embryos, knock down of proteins that mediate binding of motor proteins to organelles [3] as well as disruption of dynein’s function [7] result in impairment of centrosome centering. Previous mathematical and computational efforts attempted to model the dynamics of the centrosome driven by cytoplasmically-distributed motors. However, as detailed in the Discussion and Supporting Text S1, these models either incorrectly assume that a single motor hauls each cargo [11], or make assumptions about the reaction of motors to load that are not physical [2] and thus both lead to the conclusion that large, slow-moving cargoes are required in order to generate forces large enough to move the centrosome. Large and slow cargoes are not typical in cells, and are reminiscent of the unknown fixed intracellular structures to which motors were previously suggested to anchor to [6].

Here, we present a model for centrosome centering where the forces pulling the aster result from the cytosolic drag opposing small, fast-moving vesicles or organelles as they are hauled by molecular motors. We show that our model reproduces the observed motion of the sperm aster in large embryos such as that in *Xenopus laevis*.

## Results

**Figure 1:**
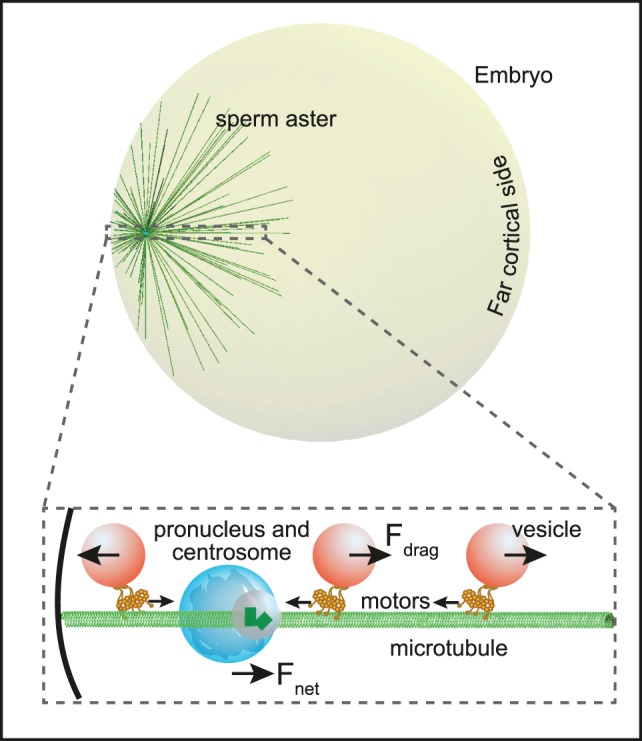
Schematic of the geometry of the embryo used in the model and the forces involved. The embryo has the spherical geometry of that of the early *Xenopus laevis* embyo with the sperm’s pronucleus and its associated microtubule aster positioned close to the cortex. As cargos are transported by minus-end directed motors towards the centrosome they experience an opposing cytosolic drag force (F_drag_, magnified schematic). That force equals the force exerted by the motors on the microtubules and points in opposite directions on the two sides of the centrosome. The net force, F_net_, on the centrosome and associated male pronucleus points towards the far cortical side since the microtubules on that side can grow longer and support more vesicles than those on the other side. The net effect is motion of the growing aster towards the cell center.

In this work we study a sperm aster centering model in which the centering force arises from the fluid drag on cytoplasmically distributed cargoes hauled by the minus-end-directed microtubule motor, dynein. As depicted in Figure 1, cargoes will experience a drag force as they move along the microtubular tracks. This force is transmitted to the microtubules by the molecular motors hauling the cargo. Since the centrosome is attached to the male pronucleus, the force ultimately acts to pull the latter. For a symmetric microtubule array, the net force would be zero. However, because microtubules elongate when not obstructed, they will be longer towards the far cortical side and thus support a larger number of moving cargoes. Hence, a net force acts on the pronucleus that pulls it towards the cell center (Figure 1). For a more detailed description of this force see the Methods. Although the forces described in our model could be responsible for centering in different cell types [3], in this work we focus on the geometry of fertilized *Xenopus laevis* embryos.

In addition to geometric constraints, the relevant centering parameters of our model are the vesicle velocities and size, microtubule polymerization rate, vesicle density on the microtubules and only the ratio of the cytoplasmic viscosity experienced by the cargo to that experienced by the centrosome and microtubules (see Methods). Of these parameters, only the average microtubule polymerization rate in *Xenopus laevis* eggs has been experimentally measured [6]. However, velocities have been measured for a variety of cargoes in different systems [20] including the *C. elegans* embryo [7] and typically range between ∼0.5 µm/s and 2 µm/s. Less is known about intracellular viscosities and values spanning several orders of magnitudes have been reported reflecting the non-Newtonian and complex nature of the cytosol [21]–[25]. However, as long as the motors hauling the cargoes are not experiencing an opposing load comparable to their stall force, the only relevant parameter is the ratio of the effective viscosities experienced by the cargoes to that experienced by the components of the sperm aster. Knowledge of the absolute values of the viscosities would be needed to quantitatively describe the sperm aster dynamics if motors were highly loaded. However, as detailed in the Discussion, the dynamics of the sperm aster will be qualitatively similar whether the motors are only slightly or highly loaded.

**Figure 2:**
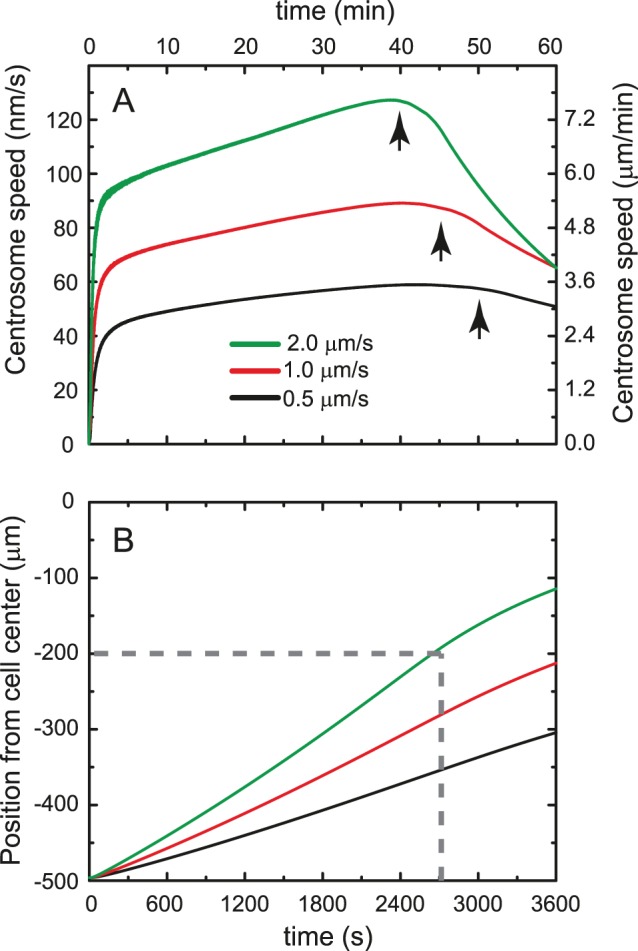
Centrosome dynamics as a function of vesicle velocity. (A) After an initial ramp up, the centrosome velocity keeps increasing at a much smaller rate. As the difference in number of vesicles moving along microtubules in the far and near cortical sides decreases, the centrosome slows down (arrows). Given that slower vesicles experience a smaller drag force, they lead to a slower centrosome. (B) The corresponding position of the centrosome shows that the small, fast-moving vesicles are sufficient to move the centrosome distances comparable to the motion of the centrosome in fertilized *Xenopus laevis* embryos. Experiments show that the centrosome moves at least 300 µm in 45 minutes; the dashed lines delineate that region. (calculation parameters: 100 microtubules; 100 nm diameter vesicles; viscosity ratio = 3; 2 vesicles/µm; 250 nm/s MT polymerization rate; vesicle velocity as indicated: 2 µm/s (green), 1 µm/s (red), 0.5 µm/s (black) in that order from top to bottom).

As shown in Figure 2A, for typical transport parameters, the centrosome motion is characterized by a quick rise of its velocity towards the center of the cell reaching ∼80% of the maximum centrosome velocity within the first 5 minutes. After the initial ramp up, the centrosome velocity keeps increasing at a much lower rate. As the centrosome approaches the cell’s center and microtubules on the far cortical side approach the cell wall, the force imbalance decreases resulting in a slowdown of the whole sperm aster. The position versus time plot shows that within the first 40–45 minutes, the centrosome moves ∼300 µm, comparable to the typical distance it moves in fertilized *Xenopus laevis* embryos [6]. In the following analysis, if the centrosome is able to move 300 µm in roughly 40–45 minutes for a particular choice of parameters, it is considered to have centered appropriately (see Methods).

### The Effect of Vesicle Velocity

Figure 2A and 2B show the velocity and position of the centrosome for vesicles moving at 0.5, 1 and 2 µm/s. In general, the faster the cargo moves, the larger the drag force it experiences, and thus the larger the force on the sperm aster. We find that for 100 nm vesicles and slow motors, i.e. 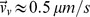, the centrosome does not center within the time window of 45 minutes observed in experiments. However, motors translocating along microtubules at four times that velocity are able to move the centrosome within that time even for these small cargoes.

### The Effect of Microtubule Density

**Figure 3:**
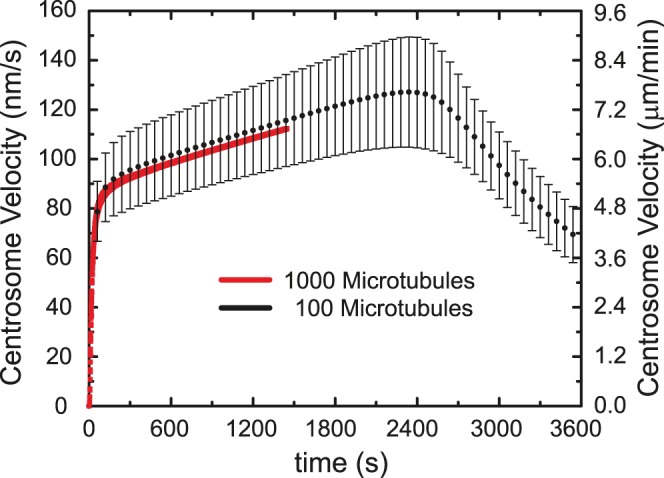
The number of microtubules comprising the aster does not alter centrosome dynamics. The randomness in the direction of the microtubules leads to variability in the dynamics. This is shown as error bars representing the standard deviation for multiple runs of the simulation for 100 MTs. The 1000 MT trace lies close to the average and within the error bars indicating similar dynamics. Although the drag arising from having more microtubules (MTs) increases with microtubule number, the number of force generating vesicles increases in the same proportion leading to identical centrosome velocity. (calculation parameters: microtubule numbers as indicated: 1000 (red), 100 (black) in order of increasing duration shown; 100 nm diameter vesicles; viscosity ratio = 3; 250 nm/s MT polymerization rate; 2 vesicles/µm; 2 µm/s vesicle velocity).

The number of microtubules comprising the aster is not experimentally known. Moreover, given that the microtubules are randomly distributed, variation in microtubule organization can result in slightly altered centrosome dynamics, as shown in Figure 3. However, we find that the average centrosome dynamics is independent of the number of microtubules used in our model. This is due to the fact that as the number of microtubules grows, the viscous drag on the aster increases, but the number of vesicles on the microtubules grows simultaneously increasing the pulling force. Hence, the aster will have the same dynamics as long as the drag facing the microtubules is significantly larger than that facing the pronucleus. The following calculations were performed with a microtubule number of 100 to accelerate computation time. To enable direct comparison of the dynamics, we used the same randomly generated microtubule organization for all calculations.

### The Effect of Cytoplasmic Viscosity

**Figure 4:**
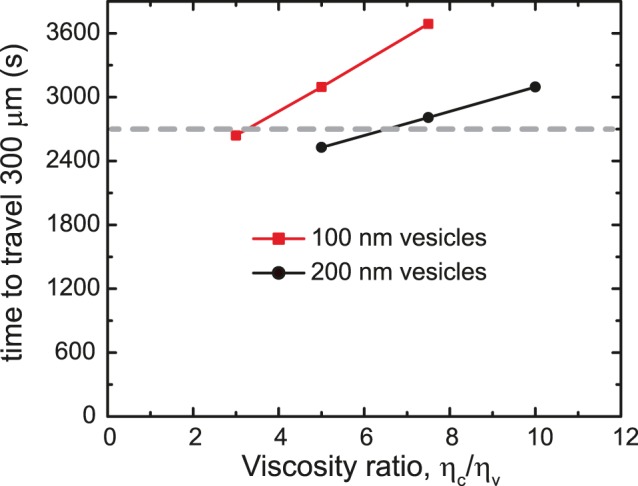
The centrosome takes longer to center for larger effective viscosity ratios. Since the aster constituents are larger than the vesicles they are also likely to experience a larger effective viscosity arising from the cell’s crowded environment. The effect of viscosity ratio on centering speed is less pronounced (smaller slope) for larger vesicles. The dashed line delineates the centering time experimentally observed. (calculation parameters: 100 microtubules; vesicle diameter: 100 nm (red squares), 200 nm (black circles); 250 nm/s MT polymerization rate; 2 vesicles/µm; 2 µm/s vesicle velocity).

The centrosome can be many times larger than the typical cargoes moved by molecular motors along microtubules. Moreover, microtubules, although thin in diameter, extend several micrometers in length throughout the cytoplasm. Because of these size differences and interference with the cytoskeletal network, it is likely that the microtubules and centrosome experience a larger effective cytoplasmic viscosity (*η_c_*) than that experienced by the cargoes (*η_v_*). As described in the Methods, our model depends on the ratio of these effective viscosities rather than their individual values; however, since neither the individual values nor ratios have been measured experimentally, we decided to study the effect of varying the viscosity ratio on aster dynamics. Figure 4 shows the effect of varying the viscosity ratio on the centering time for two different cargo diameters, 100 nm and 200 nm. As expected, the larger the viscosity experienced by the aster components compared to that experienced by the cargos, the longer it takes the aster to center. Interestingly, this effect is less pronounced for larger vesicles as seen by the smaller slope of the line for 200 nm–sized vesicles. For this vesicle size, the centering time lies within 10% of the average observed centering time over a wide range of viscosity ratios. Detailed examination of Equation 2 shows that the centrosome velocity is proportional to vesicle radius, *R_v_*, divided by the ratio of effective viscosities *(η_c_/η_v_)*. Thus, the time it takes the centrosome to move a certain distance, as plotted in Figure 4, is proportional to the viscosity ratio divided by the vesicle radius; hence the less pronounced dependence for larger vesicles.

### The Effect of Microtubule Polymerization Rate

**Figure 5:**
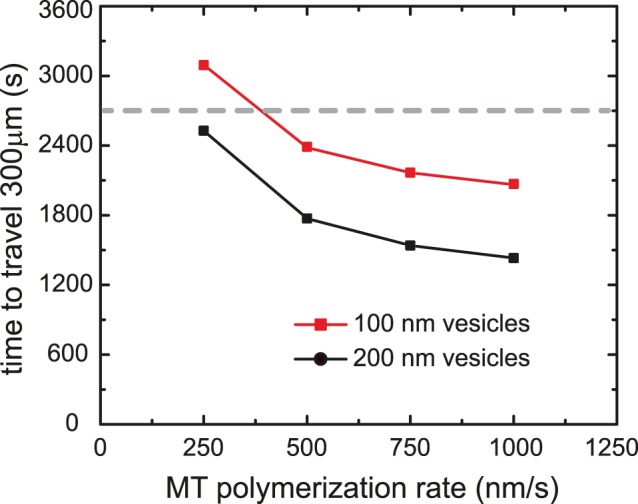
A larger microtubule polymerization rate leads to limited increase in centrosome speed. While the centrosome centers within a shorter time for moderate increase in the microtubule (MT) polymerization rate, the centering time saturates for large polymerization rates. This is due to two competing factors: a larger polymerization rate leads to a larger asymmetry in the numbers of force-generating vesicles but also leads to the microtubules touching the far cortical side sooner. Experiments show that centering is completed before the microtubules touch the far cortical side. For all polymerization rates shown except 250 nm/s, the microtubules touch the far cortical side before the centrosome reaches its central position. An average microtubule polymerization rate of 250 nm/s was reported for *Xenopus laevis* embryos. The dashed line delineates the centering time experimentally observed. (calculation parameters: 100 microtubules; vesicle diameter: 100 nm (red squares), 200 nm (black circles); viscosity ratio = 5; 2 vesicles/µm; 2 µm/s vesicle velocity).

As microtubules grow, they are able to accommodate more cargoes and thus increase the force on the centrosome. On the side closer to the cell periphery, microtubule length is limited by the cortex, however, on the far cortical side the limiting factor is the microtubule polymerization rate. The net force on the centrosome results from an excess of moving cargoes in the far cortical side, and thus would be expected to be larger for larger microtubule polymerization rate. Consistent with this, Figure 5 shows that the centering time decreases with increased polymerization rate. The figure also shows that the centering time levels off for large polymerization rates. This can be understood by considering that a very large polymerization rate implies that the centrosome reaches its maximum velocity and starts its slow-down sooner, since the microtubules on the far cortical side start touching the cortex. In Figure 5, for polymerization rates exceeding about 250 nm/s, all microtubules touch the boundaries before the aster reaches the center. Images of centering asters in *Xenopus laevis* embryos suggest that the centrosome reaches the center before the microtubules reach the boundaries on the far cortical side [6]. Intriguingly, the rate of microtubule elongation as inferred from the reported aster growth rates is 15 µm/min (250 nm/s) for which the centrosome centers before the microtubules touch the far cortical side in the simulation.

## Discussion

We described a mechanism for sperm aster centering in which the centering force arises from the hydrodynamic drag experienced by cargoes hauled by molecular motors along the microtubules comprising the aster. A net force hauls the aster towards the cell center because the microtubules on that side are longer and thus support a larger number of motor-driven cargos. Although we focused our study on sperm aster centering in fertilized *Xenopus laevis* embryos, the mechanism could be responsible for centering in other systems as well [3].

All available evidence suggests that cytoplasmic dynein, a minus-end directed motor, plays the main role in centrosome centering [1], [3], [9]. Given that the microtubules’ minus ends are at the centrosome, the force that minus-end motors apply on the microtubule will pull the aster towards the cargo resulting in a net aster transport in the direction of more cargoes. While many cargos move bidirectionally, switching direction often between minus-end and plus-end directed motion [26], [27], a recent study showed that disruption of cargo transport only in the minus end direction during centrosome centering in *C. elegans* embryos results in the centrosome failing to center [3]. This suggests that, plus-end transport, if present, does not play a significant role in centering. Plus-end motors apply a force pointing towards the centrosome, and would antagonize the centering force. However, evidence suggests that distributed loads applied by motors in the direction away from the microtubule’s free end are sufficient to cause local buckling of the microtubule [28] due to the relatively small buckling force of the microtubules [10], [16], [29]. Thus, if buckling occurs, plus-end motion of the cargoes will not transmit a force to the centrosome in large cells. On the other hand, if buckling does not occur, our model still predicts centrosome centering dynamics as long as a net bias in minus-end transport exists. In this case, the cargo density used in our model would reflect not the true minus-end directed cargo density, but rather the effective density when plus-end cargoes are accounted for. We therefore only considered minus-end moving cargoes in this work. Cargoes need not accumulate near the centrosome as minus-end excursions of bidirectionally moving cargoes could provide the same centering effect.

Describing a mechanism by which cytoplasmically distributed motors, in particular dynein, can center the centrosome has gathered significant attention in recent years [2], [3], [6], [11]. However, previous efforts to mathematically or computationally model centrosome centering required the use of an assumed force-velocity response of the dynein motors in order to find the force transmitted by the motor to the microtubule [2], [11]. This approach has its drawbacks: first, precise knowledge of the force-velocity curve is required to quantitatively describe the dynamics. A linear force-velocity curve was assumed in both studies and incorrectly implemented in [2] (see the Supporting Text S1 for a discussion of the model assumptions). However, it has been shown that motors exhibit a nonlinear force-velocity curve [30]–[34]. Second, cargoes in vivo are hauled by multiple copies of molecular motors [35], and the force-velocity curve depends on the number of motors actively hauling the cargo [36], [37]. The activity of multiple motors was overlooked in previous works resulting in an underestimation of the force each cargo can provide. These assumptions led the authors to conclude that large, and untypically slow-moving cargoes were needed to provide enough force to center the centrosome as explicitly stated in [11] and implicitly concluded from [2] as detailed in the Text S1.

Our model is not sensitive to these factors as, regardless of the number of motors hauling the cargo and the exact shape of the motors’ force-velocity relation, the force they collectively exert on the microtubule will be equal to the cytosolic drag force experienced by the cargo. To fully determine that force, one needs to know the cargo velocity, which is readily measured in living cells, and the effective cytoplasmic viscosity which we discuss further below. Using this approach, we find that small, fast-moving cargoes can generate sufficient force to center the sperm aster over distances of the order of half a millimeter. This is enough to center the sperm aster in fertilized *Xenopus laevis* embryos in the measured time-scales of 40–45 minutes. We find typical centrosome centering speeds of ∼100–200 nm/s (300–500 µm in 40–45 min) which agree with those observed experimentally [6]. Furthermore, these speeds also agree with those measured for male pronuclei centering in *C. elegans* embryos [3], suggesting that this mechanism could also be more general.

### Role of Viscosity

The cytosolic viscosities experienced by both the intracellular cargoes and by the sperm aster are important parameters of our model. On the one hand, the viscosity the cargoes experience will determine the force each cargo transmits to the microtubule. On the other hand, the viscosity experienced by the sperm aster limits its speed. Furthermore, the viscous force each cargo experiences is distributed over the number of active motors on that cargo and, because of the nonlinear force-velocity response of the motors, it will determine the velocity the cargo moves at.

The shape of the force-velocity curve can be different for different motors [30], [32]–[34], [38]. A feature common to these curves is the existence of two force regimes: one in which the motor velocity changes rapidly with the opposing force (load-sensitive regime) and another where the motor velocity changes only slightly with opposing force (load-insensitive regime). For kinesin, the load-insensitive regime extends from low forces up to about half the stall force of the motor, and the load-sensitive regime appears at high forces [34]. Dynein’s force-velocity curve is variable depending on the organism. Recent reports of experiments and simulation on mammalian dynein suggest a load-sensitive regime at low loads followed by a load-insensitive regime at high loads [32], [38]. Yeast dynein, however, exhibits a short load-insensitive regime at small loads and another at high loads [30].

Computing the precise aster dynamics for a particular system requires precise knowledge of the cytosolic drag force, the number of motors active per cargo, and the force-velocity (F–v) curve for single and multiple dynein motors hauling the cargo. To complicate matters more, the value of stall force and maximum motor velocity can differ from one biological system to another or from those measured in vitro. However, as we argue below, the aster dynamics will be qualitatively the same regardless of the shape of the force-velocity curve, of whether the motors are functioning in the load-sensitive or load-insensitive regimes, or of the value of the maximum motor velocity or force.

**Figure 6:**
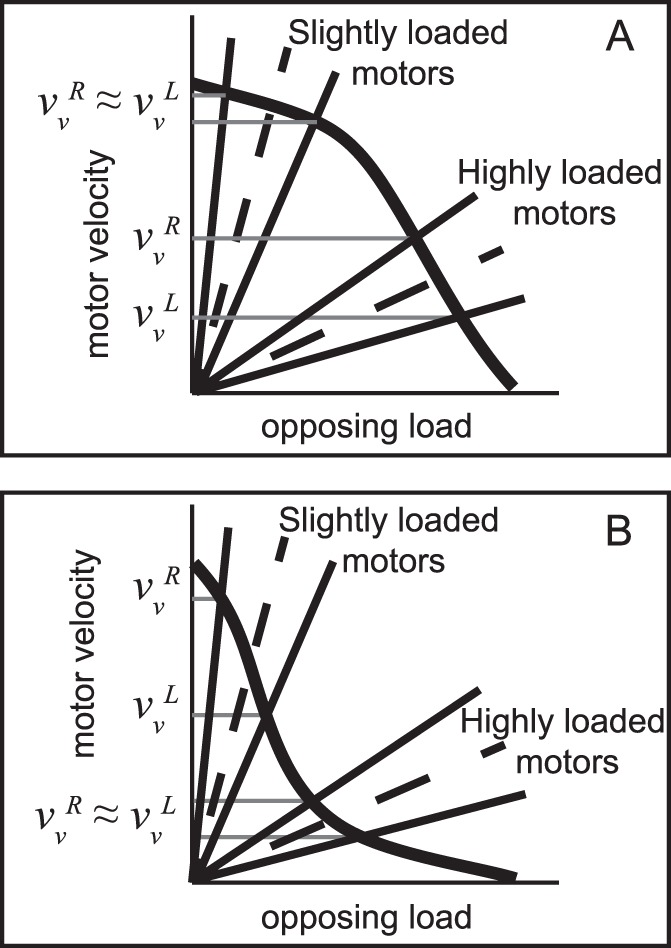
Using centrosome dynamics to study cytosolic loading of molecular motors. (A)A schematic sketch of a convex-up force-velocity (F–v) relation for a molecular motor shows that the velocity of the motor decreases only slightly up to an opposing load of about one half its stall force then decreases precipitously. A motor hauling a cargo will experience an opposing load from cytosolic drag that determines its speed at the intersection of the load line (dashed) and the F–v curve. When the aster starts moving towards the center, the minus-end motors on the far cortical side will experience a smaller load and speed up (*v*_v_^R^) while those on the other side will slow down (*v*_v_^L^) (as indicated by the two load lines). For slightly loaded motors, the motor speeds on either side of the centrosome will not differ appreciably, while they will diverge significantly for highly loaded motors. (B) Molecular motors with a concave-up force velocity curve will result in slightly-loaded motors exhibiting a large difference in cargo velocity on either side of the centrosome. Measuring cargo velocities moving along centering aster microtubules can help understand the loading state of the motors if their force-velocity relation is known.

As shown in Figure 6, for any given F–v curve, the velocity of the cargo is determined by the intersection of the motors’ force-velocity curve and the load line (dashed line). As the aster moves towards the cell center, the cargos on the half of the aster closer to the center will start experiencing a reduced cytosolic drag while those on the other side will experience a larger one (solid lines on either side of the dashed load line). If the motors are operating in a load-insensitive region of the force-velocity curve, this difference in drag force does not result in an appreciable velocity difference between the cargoes moving in the far cortical side and those in the near cortical side (slightly-loaded motors in Figure 6A and highly-loaded motors in Figure 6B). However, if the motors are operating in a load-sensitive regime of the F–v curve the motion of the aster will decrease the load on the motors hauling cargos on the half of the aster closer to the cell’s center making them move faster while those on the other side will move at a slower rate along the microtubules. This altered motion will change the magnitude, but not the direction of the net force applied to the aster. Detailed knowledge of the motor number and properties as well as the rheological properties of the cytosol would be required for a quantitative description of the ensuing aster dynamics. However, the fact that qualitatively the aster dynamics remain unaltered provides predictions from which the loading state of the motors can be deduced as detailed below.

A typical 100 nm diameter cargo driven by 4–5 load-sharing motors, each with a stall force in the range of 1–7 pN, will be moving at a velocity determined by the effective cytosolic viscosity it experiences and the shape of the force-velocity curve of the motors. However, velocities as large as 2 µm/s can be attained if the effective viscosity of the cytosol is as large as 2 Pa.s (2000 times the viscosity of water). Given that minus-end directed cargos on either side of the moving centrosome experience an additional opposing or assisting load, the ensuing velocity of the cargo will depend on the details of the force velocity curve. Our model provides a testable prediction that enables determining whether the motors are functioning in a load-sensitive or load-insensitive regime as described below. If the shape of the force-velocity curve is known, this information would be enough to determine whether motors are slightly or highly loaded.

The velocity of the cargoes as measured in the microscope (laboratory reference frame, *f* ) are 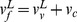 for the vesicles moving on the near cortical side (superscript *L* for ‘Left’) and 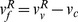 for those on the far cortical side (superscript *R* for ‘Right’), where 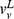 and 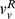 are the velocities of the motors with respect to the microtubule on the respective side of the centrosome, and 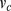 is the velocity of the centrosome. If one considers cargos moving along the line defined by the centrosome motion, then, the mathematical construction given by: 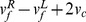 will be equal to zero if the motors are moving in a load-insensitive regime since 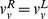, and larger than zero in a load-sensitive regime since 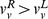 (see Figure 6). Since 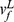, 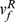 and 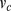
are all measurable using time lapse microscopy in many biological systems, this construction together with the shape of the force-velocity curve enable determining whether the motors are highly loaded or not.

In conclusion, the model we described provides a mechanism for centrosome centering that can still work in cases where neither microtubule pushing nor cortical motor pulling is possible. While we developed the model having the *Xenopus Laevis* embryo in mind, the results we find could be applicable to other cell types. Intriguingly, close examination of the average speed of *C. elegans* pronucleus migration reveals that it is comparable to that of *Xenopus laevis*; both move at about 7 µm/min [6], [7], [11]. We showed that such speeds are attainable through the force generated by molecular motors as they haul cargos at the typical speed of 2 µm/s. This possible ubiquity of the model can facilitate testing its predictions by choosing a system that is tractable for the experimental methods needed.

## Materials and Methods

### The Biological Model

We study the sperm aster motion in *Xenopus laevis* fertilized eggs in our computational model. For more details about the process see reference [6]. Briefly, the *Xenopus* egg has a spherical shape and measures around 1200 µm in diameter. Upon fertilization, the male pronucleus and centrioles that form the microtubule organizing center enter the egg on the animal pole. This is known as the sperm aster. As microtubules grow, the sperm aster grows and moves towards the center of the cell; this process takes about 45 minutes. The diameter of the sperm aster has been observed to grow at about 30 µm/s. The sperm aster does not always reach the center, but in most cases travels at least 300 µm. The sperm aster then disintegrates and the mitotic spindle is formed for the cell to undergo the first cleavage division. Here, we only consider the sperm aster centering process that takes place right after fertilization. In the following, when the centrosome is referenced, it is understood that both the male pronucleus and the centrosome move together.

### The Physical Model

A schematic of the relevant forces is depicted in Figure 1. Vesicles moving through the cytoplasm via molecular motors experience a drag force given by: 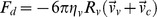, where *η_v_* is the cytoplasmic viscosity experienced by the vesicle, *R_v_* is the vesicle radius and 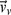 and 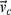 are the velocity of the vesicle with respect to the microtubule and the velocity of the centrosome, respectively. The velocity of the vesicle relative to the cytoplasm, 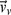
+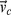, is the relevant quantity for the drag force. At low Reynolds numbers, which is the relevant regime for cargo transport, the force transmitted to the microtubule by the motors hauling a single cargo will equal the drag force the cargo experiences. The force applied by the motors moves the sperm aster through the cytoplasm. We consider the male pronucleus and centrosome together as a solid sphere, of radius *R_c_* for which the drag force is given by: 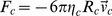 and the microtubules as thin, rigid, cylinders of radius *a* and length *L*, respectively, with the drag force on each filament given by slender-body theory [39], [40]:
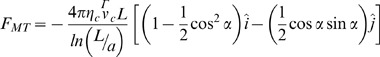
(1)


where α is the angle the microtubule makes with the direction of motion, being the line connecting the centrosome to the cell center in our case (see below). To calculate the drag on the aster, we consider each microtubule as a thin, long rod moving through the fluid instead of considering the whole aster as a solid sphere since experimental observations have shown that the centrosome moves at a relatively small speed (∼100–200 nm/s) [6], suggesting that the cytosolic fluid can flow through the aster and not just around it. It is important to note that under these conditions, the net drag force on the aster is much larger than that on an “effective” sphere with a radius equal to the length of a microtubule, as was considered in Reference [2]. Since the microtubules of the sperm aster extend over a large volume, they are likely to experience a higher effective cytosolic viscosity than that experienced by small vesicles (∼100–500 nm). This is mainly due to the crowding and cytoskeletal content of the cytoplasm, actin and intermediate filaments, which results in the medium being non-Newtonian with a size- and rate-dependent viscosity [24]. Since the aster moves relatively slowly (∼100 nm/s), and given that a detailed description of the rheological properties of the cytoplasm is unknown, here we use two effective viscosities: one for the vesicles (*η_v_*) and a larger one for the centrosome and microtubules (*η_c_*). As is shown below in the force balance equation (Eq. 2), only the ratio of the viscosities (*η_c_/η_v_*), and not their individual absolute values, determines the sperm aster dynamics in our model. The force balance equation is:

**net drag force on vesicles = drag force on centrosome+drag force on microtubules**


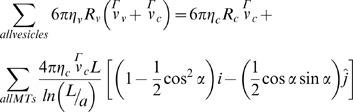
(2)

The term on the left-hand side of equation 2 is the net drag force acting on the vesicles as they move along the microtubules through the cytoplasm. Vesicles on the far cortical side moving towards the centrosome experience a smaller drag force per vesicle than those on the near cortical side since 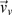 points in the opposite direction to 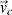 for the former but not for the latter. However, given that the microtubules on the far cortical side are longer, they will support more vesicles. As long as the collective force of these vesicles is larger than that produced by the vesicles on the near cortical side, the centrosome will move towards the center as observed in experiments. This is the scenario investigated in this work. Only motion along the line connecting the centrosome to the cell center is considered in this work as the net force in other directions is zero, on average, due to the symmetry of the geometry used.

### Computational Model and Physical Parameters

The cell boundary is defined as a sphere measuring 1000 µm in diameter in our simulation. The initial position of the centrosome is 4 µm away from the cell wall in the equatorial plane. Given the symmetry of the simulated embryo, we only consider centrosome motion in that plane as there is no off plane motion on average. The imaging plane in experiments is above the embryo equator in the animal pole [6], and some off-plane motion is possible. The centrosome together with the pronucleus are defined as a sphere with a radius of 2 µm. Microtubules are randomly generated and isotropically distributed around the centrosome and their initial length is set to 2 µm. We do not include microtubule dynamic shrinking and growth, or catastrophe and only consider the average growth rate of aster microtubules (15 µm/min), as that is reported from experiments [6]. Individual microtubule dynamics are likely to introduce short time stochasticity into the process, but will not alter the average behavior which is the focus of this work. A microtubule will stop growing if it is touching the cell boundary. Vesicles and organelles hauled over long distances along microtubules range in size, and typical cargoes have diameters from about 100 to 1000 nm [20], [35], [41]–[45]. As Equation 2 shows, the force resulting from the motion of each cargo scales linearly with its diameter. Here, we focus on the lower end of the vesicle size range. Smaller cargoes will provide a smaller force per cargo to the centrosome and thus serve to test the conditions under which that lower limit is sufficient to reproduce the experimentally observed motion and corresponding time scales. Similarly, we also fix the density of vesicles on the microtubules to 2 vesicles/µm. Given the lack of experimental data we chose this small value to test the limits of the model since the force scales with the density as inferred from Equation 2. Motor velocities with respect to the microtubule are varied between 0.5 µm/s to 2 µm/s, however, for each calculation a single value was used for all the cargos to get the average behavior. These values were chosen to span the range of experimentally observed parameters in a myriad of transport systems [20]. To obtain the dynamics of centrosome centering, equation 2 was solved for the centrosome velocity at every time step of 0.5 seconds. The centrosome position was updated by integrating the centrosome velocity over that time step.

## Supporting Information

Text S1. (PDF)
