# Supplementary material for: Cargo Transport by Cytoplasmic Dynein Can Center Embryonic Centrosomes
Source: PLoS One. 2013 Jul 1;8(7):e67710. doi: 10.1371/journal.pone.0067710 (PMC3698173; doi:10.1371/journal.pone.0067710)
Supplement: Text S1. — (PDF) [file pone.0067710.s001.pdf]

Supporting Text S1

Cargo transport by cytoplasmic dynein can center embryonic centrosomes

Rafael A. Longoria and George T. Shubeita

Previous attempts at modeling centering force via cytoplasmically-driven cargoes

Forces due to cytoplasmically moving cargoes have been hypothesized as a possible mechanism for centrosome centering in the past (1-3). Conceptually, this mechanism is simple: a cargo moving through the cytoplasm will experience a drag force opposite to its direction of motion. The motors pulling it generate an equal force that is transmitted to the substrate, i.e. the microtubule, and thus pull the microtubule and associated structures in the direction of the force. Although this mechanism has been conceptually proposed for centrosome centering, attempts to mathematically analyze its consequences in the context of the C. elegans embryo lead to a conclusion that contradicts its premise: to support the centering force, the cargoes have to be either stationary or large and moving only slowly. The need for slow cargos was explicitly stated in (3). Kimura and Onami implicitly reach the same conclusion (1). Careful analysis of the latter work's assumptions readily show that in their model, slow moving cargoes are responsible for the centering force acting on the centrosome as discussed below. Whether the authors were aware of this or not is not of our knowledge. In contrast our approach to analyze this simple conceptual model leads us to conclude that the typical, fast-moving and small cargos are sufficient to generate the forces required to center the centrosome within the experimentally observed timescale for the *Xenopus* embryo. In the following, we detail the crucial differences between our model and those published previously, and discuss how the inaccurate assumptions made in those attempts lead to erroneous conclusions.

Force-generating equations

There are two ways to find the force exerted on a MT by molecular motors: 1) directly use the force-velocity curve for each motor to determine the force corresponding to its velocity; and 2) indirectly calculating the force by finding the drag force the cargo experiences as it is hauled through the cytoplasm; this force will be equal to the force the motors apply to move it (see the main text for more detail). Previous attempts at modeling centrosome centering via cytoplasmically moving cargoes have used the force-velocity curve for a single motor to calculate the force on a given MT (1, 3). This assumption is incorrect since it is well known that individual cargoes *in vivo* are typically hauled by multiple motors (4, 5). As discussed further below, assuming single motors move cargoes led to the inconsistent conclusion that very slow and large cargoes are needed. In the work of Kimura and Onami, the physical equations used to account for the force-velocity curve lead to physically unreasonable motor behavior. In the following we discuss their model and its implications.

In order to calculate the force exerted by each cargo, the authors defined the following system of reference: A microtubule (MT) was defined by a direction vector $\hat{u}$ that points from the minus-end towards the plus-end. Thus, MTs pointing towards the near cortical side will have at least one component of their direction vector pointing in opposite direction to those MTs pointing towards the far cortical side. The motor speed was calculated by the dot product between the direction vector of the MT in which the motor is moving and the velocity vector of the pronucleus. Finally, the force exerted by the motor on the MT was calculated by choosing between 3 possible states depending on the value of the motor speed:

$$F_{pull} = \begin{cases} D \cdot L \cdot F_{stall} & \left(\vec{V}_{nuc} \bullet \hat{u}\right) \leq 0 \\ D \cdot L \cdot F_{stall}\left[1 - \left(\vec{V}_{nuc} \bullet \hat{u}\right)/V_{\max}\right] & \left(0 < \vec{V}_{nuc} \bullet \hat{u} \leq V_{\max}\right) \\ 0 & \left(\vec{V}_{nuc} \bullet \hat{u} > V_{\max}\right) \end{cases} \quad \text{Equation 3}$$

The first force value states that when $\vec{V}_{nuc} \bullet \hat{u} \leq 0$ motors carrying a cargo will exert their maximal force (stall force) on the microtubule. The second value represents the force-velocity curve chosen (linear relation). The last force value states that when the motor's substrate moves faster than the motor maximum velocity, the motor cannot exert a force on the MT. This expression for the force is incorrect for two reasons. First, it implies that motors are stationary with respect to the cytoplasmic fluid. Not only is this assumption restrictive, but it is inconsistent with the model's idea of cytoplasmically moving cargoes generating the pulling forces that

center the pronucleus. Shinar *et al.* corrected for this assumption by using the relative velocity of the motor with respect to the fluid (3). Second, careful analysis of the three conditions shows a much more restrictive and unrealistic behavior. For example, for a MT pointing towards the far cortical side, its direction vector will have a component along the direction of motion of the pronucleus, and thus $\vec{V}_{nuc} \bullet \hat{u} = |\vec{V}_{nuc}| > 0$, satisfying the second condition in equation 3 and generating a force depending on the particular value of $\vec{V}_{nuc}$; however, for a MT pointing towards the near cortical side, this same dot product gives $\vec{V}_{nuc} \bullet \hat{u} = -|\vec{V}_{nuc}| < 0$, satisfying the first condition in equation X. This condition explicitly sets the motor force to its maximum force value, known as the motor stall force. Therefore, in the model by Kimura and Onami, motors on the far cortical side will exert a force given by the force-velocity curve, but motors on the near cortical side are always exerting their maximal force. Because at their stall force, by definition, the motor velocity is zero, this implies that motors on the near cortical side are stationary with respect to the MTs. If the pronucleus is being dragged along with its associated centrosome and MTs, motors at rest with this structure will be dragged along at the same velocity of the pronucleus. Using values used in that work ($\eta \approx 1 Pa \cdot s, V_{nuc}^{\max} \approx 250 nm/s$), the maximum drag force on a vesicle of about 200nm in diameter moving with the pronucleus is:

$$F_{drag}^{\max} = 6\pi\eta R V_{nuc}^{\max} \approx 0.5 pN$$

This value is smaller than the stall force of the motors used in their work (1.1pN). Therefore, even if the pronucleus is moving at about its maximally experimentally observed centering speed, cargoes will not experience sufficient drag forces to fully stop, yet the force equation they use forces the motors to stop.

In summary, the force equation used by Kimura and Onami is neither correct nor consistent with the model itself, thus the ensuing dynamics of the centrosome needs revisiting.

Previous models require slow moving cargoes

Shinar *et al.* found that their model required large, slow moving cargoes in order to generate sufficiently large forces to center the pronucleus (3). Kimura and Onami did not look at the typical cargo velocity predicted by their model for pronucleus centering to take place. However, this velocity can be estimated from the values reported in their work. The motor velocity is defined as $v = \vec{V}_{nuc} \bullet \hat{u}$, thus the maximum velocity this expression can take is the pronucleus

velocity $\vec{V}_{nuc}$. In *C. elegans*, the male pronucleus velocity is about 250nm/s. Thus, the model requires that cargoes move at velocities smaller than 250 nm/s to generate the centering forces. Cargo velocities have been measured in a plethora of systems and in a large number of these (including *C. elegans*), cargo velocities exceed 1µm/s. Indeed, in experiments performed by the same authors, centering was attributed to forces mediated by fast cargoes in the *C. elegans* embryo (2). To our knowledge, our work presented in the main text is the only one that demonstrates that fast moving cargoes (> ~1µm/s) are required to generate sufficiently large centering forces to position the centrosome within the experimentally observed length and time scales. This was only possible using a different approach than previously attempted: considering the drag forces on the cargoes rather than the force-velocity curve of the cargoes.

The shape of the force-velocity curve and the number of active motors

Force-velocity curves for molecular motors have been reported to have various shapes (concave up, concave down, linear, etc.(6-9)). Previous works have assumed a linear F-v curve, under the argument that this shape is representative enough of the behavior of a motor (mainly that it slows down with increasing opposing force). Furthermore, they use this F-v curve to model the velocity and/or the force a motor transmits to the microtubule. Although using the F-v curve to model motor behavior is in principle correct, it can lead to underestimation of the velocity at which a given cargo moves at if used incorrectly. For example, it has been shown that many intracellular cargoes are hauled by multiple copies of molecular motors, and the load the cargo faces is distributed over all the active motors at any given time (5, 10). In this case, F-v curves would need to be scaled up or down according to the number of motors active on each cargo. Previous works did not account for this, and in essence are one-motor models thus leading to the underestimation of the velocity the cargoes move at (1, 3). Our work circumvents this pitfall by focusing instead on the behavior of the cargo directly, and not that of the motors. Regardless of the number of active motors on a given cargo, the drag force experienced by the latter is proportional to its size, velocity and cytoplasmic viscosity. Since this force is provided by all the motors active on the cargo, the force transmitted to the microtubule is identical to the drag force on the cargo. This approach does not require making assumptions about the properties of the motors, and instead allows us to use experimentally observed values for cargo velocities to test whether small, fast moving cargoes can generate sufficiently large forces to center the pronucleus.

Supplementary References

1. Kimura A & Onami S (2005) *Computer Simulations and Image Processing Reveal Length-Dependent Pulling Force as the Primary Mechanism for C. elegans Male Pronuclear Migration Developmental Cell* **8:** 765-775.
2. Kimura K & Kimura A (2011) *Intracellular organelles mediate cytoplasmic pulling force for centrosome centration in the Caenorhabditis elegans early embryo Proc Natl Acad Sci U S A* **108:** 137-142.
3. Shinar T, Mana M, Piano F, & Shelley MJ (2011) *A model of cytoplasmically driven microtubule-based motion in the single-celled Caenorhabditis elegans embryo Proceedings of the National Academy of Sciences of the United States of America* **108:** 10508-10513.
4. Gross SP, Vershinin M, & Shubeita GT (2007) *Cargo transport: two motors are sometimes better than one Curr Biol* **17:** R478-486.
5. Shubeita GT & Gross SP (2012) in *Comprehensive Biophysics*, ed. Egelman EH (Elsevier).
6. Gennerich A, Carter AP, Reck-Peterson SL, & Vale RD (2007) *Force-induced bidirectional stepping of cytoplasmic dynein Cell* **131:** 952-965.
7. Rai AK, Rai A, Ramaiya AJ, Jha R, & Mallik R (2013) *Molecular adaptations allow dynein to generate large collective forces inside cells Cell* **152:** 172-182.
8. Toba S, Watanabe TM, Yamaguchi-Okimoto L, Toyoshima YY, & Higuchi H (2006) *Overlapping hand-over-hand mechanism of single molecular motility of cytoplasmic dynein Proceedings of the National Academy of Sciences of the United States of America* **103:** 5741-5745.
9. Visscher K, Schnitzer MJ, & Block SM (1999) *Single kinesin molecules studied with a molecular force clamp Nature* **400:** 184-189.
10. Leidel C, Longoria RA, Gutierrez FM, & Shubeita GT (2012) *Measuring molecular motor forces in vivo: implications for tug-of-war models of bidirectional transport Biophys J* **103:** 492-500.
